# Antibacterial Effect and Mode of Action of Flavonoids From Licorice Against Methicillin-Resistant *Staphylococcus aureus*

**DOI:** 10.3389/fmicb.2019.02489

**Published:** 2019-11-05

**Authors:** Shuai-Cheng Wu, Zhi-Qiang Yang, Fei Liu, Wen-Jing Peng, Shao-Qi Qu, Qian Li, Xiang-Bin Song, Kui Zhu, Jian-Zhong Shen

**Affiliations:** ^1^Beijing Advanced Innovation Center for Food Nutrition and Human Health, College of Veterinary Medicine, China Agricultural University, Beijing, China; ^2^College of Veterinary, Qingdao Agricultural University, Shandong, China; ^3^College of Agriculture and Forestry, Linyi University, Shandong, China

**Keywords:** flavonoids, glabrol, MRSA, mode of action, licorice

## Abstract

*Staphylococcus aureus* is a bacterial pathogen that causes food poisoning, various infections, and sepsis. Effective strategies and new drugs are needed to control *S. aureus* associated infections due to the emergence and rapid dissemination of antibiotic resistance. In the present study, the antibacterial activity, potential mode of action, and applications of flavonoids from licorice were investigated. Here, we showed that glabrol, licochalcone A, licochalcone C, and licochalcone E displayed high efficiency against methicillin-resistant *Staphylococcus aureus* (MRSA). Glabrol, licochalcone A, licochalcone C, and licochalcone E exhibited low cytotoxicity without hemolytic activity based on safety evaluation. Glabrol displayed rapid bactericidal activity with low levels of resistance development *in vitro*. Meanwhile, glabrol rapidly increased bacterial membrane permeability and dissipated the proton move force. Furthermore, we found that peptidoglycan, phosphatidylglycerol, and cardiolipin inhibited the antibacterial activity of glabrol. Molecular docking showed that glabrol binds to phosphatidylglycerol and cardiolipin through the formation of hydrogen bonds. Lastly, glabrol showed antibacterial activity against MRSA in both *in vivo* and *in vitro* models. Altogether, these results suggest that glabrol is a promising lead compound for the design of membrane-active antibacterial agents against MRSA and can be used as a disinfectant candidate as well.

## Graphical Abstract

Glabrol isolated from licorice rapidly kill MRSA *via* the disruption of the membrane permeability and the proton motive force.

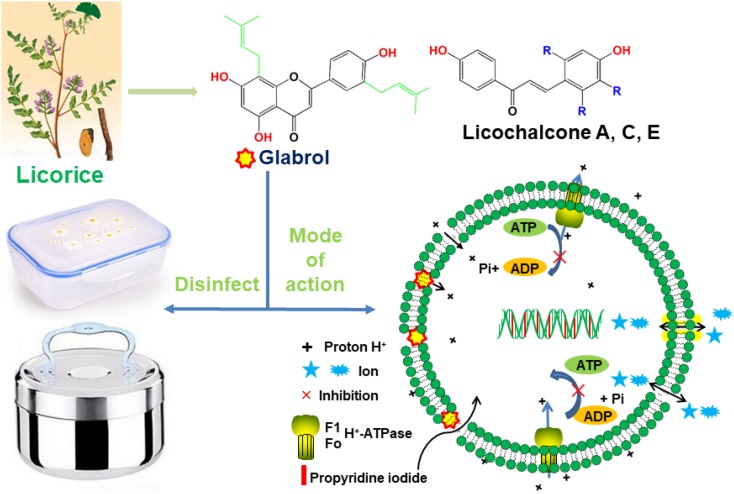

## Introduction

Bacterial foodborne diseases is a major public health concern worldwide. 9.4 million illnesses and 1,200 deaths are caused by foodborne bacterial pathogens every year in the United States (Kadariya et al., 2014). *Staphylococcus aureus* is a highly virulent pathogen that causes food poisoning, skin and soft tissue infection, and sepsis (Claeys et al., 2019; Necidova et al., 2019; Packer et al., 2019). The disinfection of cooking utensils and food storage environments is important for the prevention of foodborne diseases due to cross-contamination during food preparation (Chang et al., 2017). Moreover, the emergence and spread of multidrug resistant *S. aureus* isolates such as methicillin-resistant *S. aureus* (MRSA), further worsens the control of *S. aureus* associated infections. The number of deaths caused by MRSA are more than those caused by acquired immune deficiency syndrome (AIDS), tuberculosis, and viral hepatitis (Lee et al., 2018). Effective strategies and new antibacterial agents against *S. aureus* are therefore urgently needed (Aziz et al., 2015).

Currently, many medicinal plants and their components have been used to treat infectious diseases (Wu et al., 2016, 2018), such as *Coptis chinensis* Franch. (Yu et al. 2005), and *Rhodomyrtus tomentosa* (Zhao et al., 2019). Licorice, the root and rhizome of *Glycyrrhiza* spp., is widely used in prescriptions of traditional Chinese medicine, food, and cosmetics industry (Vaya et al., 1997). It contains many bioactive constituents, such as flavonoids, saponins, and coumarins (Wijesundara and Rupasinghe, 2019). The flavonoids isolated from licorice exhibit antibacterial activity, antioxidation activity, antiarrhythmia activity, etc. (Luo et al., 2019). In this study, the antibacterial activities of flavonoids from licorice against pathogenic bacteria, including MRSA, their possible modes of action, and potential applications were studied.

## Materials and Methods

### Antibacterial Properties

The minimum inhibitory concentration (MIC) and minimum bactericidal concentration (MBC) were determined with the broth dilution method as described by the Clinical & Laboratory Standards Institute 2017 (Wu et al., 2019). Liquiritigenin, glabrol, licoflavone A, licoflavone B, licoflavone C, licoisoflavone A, glabrene, glycyrrhisoflavone, and licochalcone A-E were purchased from Chengdu Biopurify Phytochemicals Ltd., with a purity of ≥95% (Chengdu, China). Briefly, the tested chemicals were diluted with Mueller Hinton Broth (MHB) in 96-well concave bottom plates. Bacteria were adjusted to obtain a bacterial suspension with approximately 1 × 10^6^ colony forming units (CFUs)/mL with MHB and then added into 96-well concave bottom plates (Pupo et al., 2017). To confirm the antibacterial activity of the tested chemicals, the antibacterial activity was also analyzed with Oxford cup plate methods (Aziz et al., 2016). Moreover, the constituents of the bacterial wall and membrane were used to screen for potential antibacterial targets.

### Mammalian Cytotoxicity Assay

HepG2 and Vero cells (4 × 10^4^ cells/well) were seeded into a 96-well plate and then treated with different concentrations of glabrol (1–64 μg/mL) at 37°C. After 24 h, the cells were incubated with new medium containing Cell Proliferation Reagent WST-1 (Roche, Basel, Switzerland) for 30 min, and the absorbance was measured at 450 nm. The half maximal inhibitory concentration (IC_50_) was defined as the concentration at which 50% cell viability was inhibited.

### Hemolysis Assay

Sheep red blood cells (RBC) were washed with phosphate buffered saline (PBS) twice and then diluted with PBS to obtain an 8% suspension of RBC. The 8% suspension of RBC was then mixed with different concentrations of glabrol or Triton X-100 (0.2% v/v) on a sterile 96-well plate at 37°C. After incubation for 1 h, the supernatants were harvested by centrifugation and transferred to a new 96-well plate. The absorbance was determined at 576 nm. The half hemolytic dose (Hly_50_) was defined as the concentration at which 50% RBC were lysed (Lin et al., 2017).

### Time-Kill Assay

MRSA T144 and methicillin-sensitive *S. aureus* (MSSA) ATCC29213 at the exponential phase were adjusted in MHB to obtain a bacterial suspension of approximately 1.0 × 10^6^ CFUs/mL and then treated with glabrol (4–16 μg/mL), daptomycin (8 μg/mL) + CaCl_2_ (50 μg/mL), and vancomycin (8 μg/mL) at 37°C. The bacteria were removed from the cell cultures at 1, 3, 6, 9, and 12 h serially diluted with PBS, and plated onto MHA plates. After incubation at 37°C for 24 h, the colonies were counted, and the CFUs/mL of total bacteria was calculated (Benedetto Tiz et al., 2018).

### Proton Motive Force Assay

*S. aureus* ATCC29213 cells were washed three times with buffer containing 5 mM HEPES and 20 mM glucose at pH 7.2, adjusted to obtain a bacterial suspension with densities equivalent to a 0.5 McFarland turbidity standard, and then incubated with 1 μM 3,3′-dipropylthiadicarbocyanine iodide [DiSC3(5); Thermo Fisher Scientific, Waltham, MA, United States] at 37°C for 10 min. After incubation, the *S. aureus* cells were treated with glabrol (1–16 μg/mL), daptomycin (8 μg/mL) + CaCl_2_ (50 μg/mL), vancomycin (8 μg/mL), and lysostaphin (8 μg/mL) for 50 min, and the fluorescence of the DiSC3(5) dye was monitored every 2 min for 50 min using a Tecan Infinite 200 Pro Microplate Reader at an excitation wavelength of 622 nm and an emission wavelength of 670 nm (Farha et al., 2013).

### Membrane Permeability Assay

*S. aureus* ATCC29213 cells were washed three times with buffer containing 5 mM HEPES and 20 mM glucose at pH 7.2, adjusted to obtain a bacterial suspension with densities equivalent to a 0.5 McFarland turbidity standard, and then incubated with 7.5 μg/mL propidium iodide (PI; Thermo Fisher Scientific, Waltham, MA, United States) at 37°C for 10 min. After incubation, the *S. aureus* cells were treated with glabrol (1–16 μg/mL), vancomycin (8 μg/mL), daptomycin (8 μg/mL) + CaCl_2_ (50 μg/mL), and lysostaphin (8 μg/mL) for 50 min, and the fluorescence intensity of the PI dye was monitored every 2 min for 50 min using a Tecan Infinite 200 Pro Microplate Reader at an excitation wavelength of 535 nm and an emission wavelength of 615 nm (Lin et al., 2017).

### ATP Assay

*S. aureus* ATCC29213 cells were washed three times with PBS, adjusted to obtain a bacterial suspension with densities equivalent to a 0.5 McFarland turbidity standard, and then incubated with glabrol (1–16 μg/mL) at 37°C for 60 min. After incubation, *S. aureus* cells were centrifuged at 10,000 r/min for 10 min. After centrifugation, the supernatant was harvested to detect the extracellular ATP levels and the precipitation was lysed to detect the extracellular ATP levels with the Enhanced ATP Assay Kit (Beyotime, Shanghai, China) (Lin et al., 2017).

### Reactive Oxygen Species (ROS) Assay

*S. aureus* ATCC29213 cells were washed three times with buffer containing 5 mM HEPES and 20 mM glucose at pH 7.2, adjusted to obtain a bacterial suspension with densities equivalent to a 0.5 McFarland turbidity standard, and then incubated with 10 μM dichloro-dihydro-fluorescein diacetate (DCFH-DA; Beyotime, Shanghai, China) at 37°C for 30 min. After incubation, the *S. aureus* cells were treated with different concentrations of drugs for 60 min, and the fluorescence intensity of the DCFH-DA dye was monitored using a Tecan Infinite 200 Pro Microplate Reader at an excitation wavelength of 488 nm and an emission wavelength of 525 nm (Wang et al., 2019).

### Confocal Microscopy Assay

*S. aureus* ATCC29213 cells were washed three times with PBS and then incubated with glabrol and lysostaphin for 30 min. After incubation, bacterial cells were washed with PBS and then stained with 5 μM PI and 10 μM SYTO^TM^ 9 Green Fluorescent Nucleic Acid Stain (SYTO 9; Thermo Fisher Scientific, Waltham, MA, United States). The membrane integrity of *S. aureus* was observed with a confocal fluorescence microscope (Leica, Germany).

### Molecular Docking

Discovery Studio Client 2018 was used to predict the most likely modes of binding of phosphatidylglycerol (PG), lysyl-phosphatidylglycerol (Lysyl-PG), and cardiolipin (CAL) with glabrol by receptor-ligand interaction section with the CDOCKER module. The interaction energy and types of bonding between glabrol and PG, lysyl-PG, or CAL were also calculated (Rampogu et al., 2018).

### Resistance Study

The initial MIC values of glabrol and oxacillin against *S. aureus* ATCC29213 were determined as described above. *S. aureus* ATCC29213 cells were cultured in MHB with glabrol and oxacillin at 0.5 × MIC at 37°C. After incubation for 24 h, the bacteria were incubated with new MHB with glabrol or oxacillin. The process was repeated for 30 passages (Lin et al., 2017).

### *In vivo* Assay

MRSA T144 cells were washed twice with PBS and then diluted with PBS to obtain a bacterial suspension containing 1 × 10^7^ CFUs/mL. *Galleria mellonella* larvae (∼250 mg) were randomly distributed into six experimental groups (*n* = 10/group) and then infected by injection of MRSA T144 (1 × 10^5^ CFUs/larva) into the last left proleg. At 30 min postinfection, the larvae were treated with glabrol (10, 20, 40, and 80 mg/kg), vancomycin. To investigate the possible toxicity of glabrol, the larvae were treated with glabrol (10, 20, 40, and 80 mg/kg) without infection. Larvae were stored in petri dishes in the dark at 37°C for 5 days, and the survival rate was calculated (Gibreel and Upton, 2013). The protocol was approved by the Ethical Committee for Institutional Animal Use and Care of China Agricultural University.

### Disinfection Efficacy of Glabrol Against MRSA on Cooking Utensils

MRSA T144 cells were washed three times with water and then resuspended in water to obtain a bacterial suspension of approximately 1.0 × 10^5^ CFUs/mL. Lunch boxes were sterilized with 70% ethanol, and then were infected with 1.0 × 10^5^ CFUs/mL MRSA T144 in water at room temperature. After incubation for 30 min, each region was treated with glabrol or 10% ethanol. At the indicated time, the bacteria were recovered, plated onto MHA plates, and incubated at 37°C for 24 h (Chang et al., 2017).

### Statistical Analysis

Data are presented as the means ± SD. Data were analyzed by GraphPad Prism 7 (GraphPad Software, San Diego, United States) to determine the least significant differences (*P* < 0.05).

## Results

### Antibacterial Activity of Flavonoids From Licorice

**FIGURE 1:**
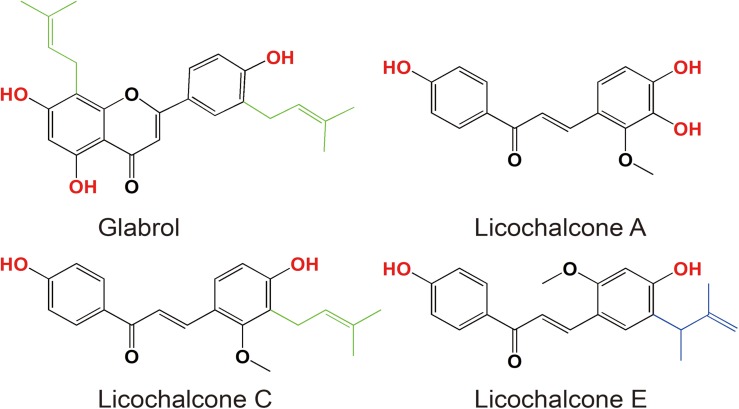
Chemical structures of glabrol, licochalcone A, licochalcone C, and licochalcone E.

**FIGURE 2:**
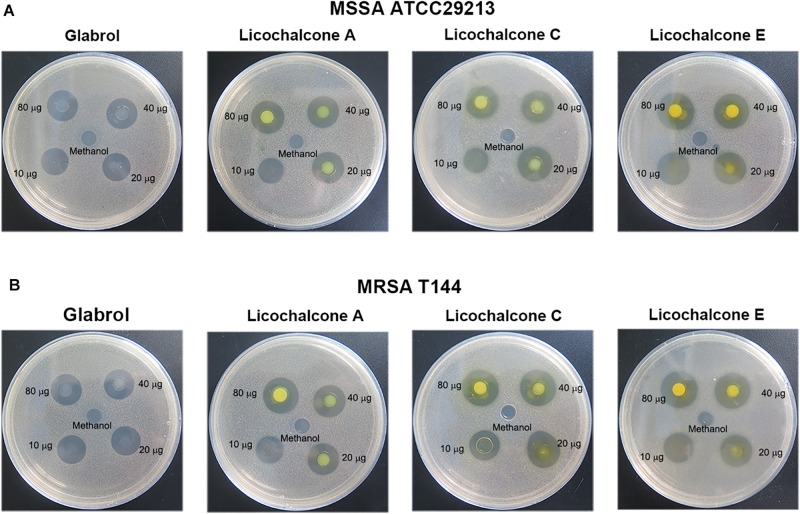
Antibacterial activity of glabrol, licochalcone A, licochalcone C, and licochalcone E against *S. aureus*. **(A)** Inhibition zones of glabrol, licochalcone A, licochalcone C, and licochalcone E against MSSA ATCC29213. **(B)** Inhibition zones of glabrol, licochalcone A, licochalcone C, and licochalcone E against MRSA T144.

The antibacterial activity of flavonoids from licorice against *S. aureus* and *E. coli* was investigated. As shown in Table 1, glabrol, licochalcone A, licochalcone C, and licochalcone E displayed high efficiency against MSSA ATCC29213 and MRSA T144 but not against *E. coli* (Figure 1). Liquiritigenin, licoflavone A, licoflavone B, licoflavone C, licoisoflavone A, glabrene, glycyrrhisoflavone, licochalcone B, and licochalcone D displayed poor efficiency against MSSA ATCC29213, MRSA T144, *E. coli* ATCC25922, and *E. coli* B2. Moreover, the MIC_90_ and MIC_50_ of glabrol, licochalcone A, licochalcone C, and licochalcone E against 20 MRSA strains were 2 and 4 μg/mL, 4 and 8 μg/mL, 8 and 16 μg/mL, 4 and 8 μg/mL, and 4 and 8 μg/mL, respectively (Table 2). In addition, the antibacterial activity of glabrol, licochalcone A, licochalcone C, and licochalcone E against MRSA T144 and MSSA ATCC29213 was further confirmed by the Oxford cup method (Figure 2).

**TABLE 1:** MICs of flavonoids from licorice (μg/mL).

**Chemicals**	**MSSA ATCC29213**	**MRSA T144**	***E. coli* ATCC25922**	***E. coli* B2**
Liquiritigenin	>128	>128	>128	>128
Glabrol	2	2	>128	>128
Licoflavone A	>128	>128	>128	>128
Licoflavone B	16	32	>128	>128
Licoflavone C	32	64	>128	>128
Licoisoflavone A	32	32	>128	>128
Glabrene	16	16	>128	>128
Glycyrrhisoflavone	32	32	>128	>128
Licochalcone A	2	4	>128	>128
Licochalcone B	128	16	>128	>128
Licochalcone C	4	4	>128	>128
Licochalcone D	32	16	>128	>128
Licochalcone E	4	4	>128	>128

**TABLE 2:** MICs of flavonoids from licorice against MRSA (μg/mL).

**Strains**	**Glabrol**	**Licochalcone A**	**Licochalcone C**	**Licochalcone E**	**Vancomycin**
MRSA T144	2	4	4	4	2
MRSA 1518	1	4	4	4	4
MRSA 1530	1	4	8	4	8
MRSA CT-1	2	2	4	1	8
MRSA CT-2	4	4	4	4	8
MRSA CT-3	2	2	16	16	4
MRSA CT-4	2	1	2	0.5	4
MRSA CT-5	2	1	4	2	4
MRSA CT-6	2	2	4	4	4
MRSA CT-8	2	4	8	8	4
MRSA CT-10	2	2	4	4	4
MRSA HN1	2	4	1	1	8
MRSA HN2	1	2	2	4	4
MRSA HN3	1	2	2	2	8
MRSA HN4	4	4	16	8	16
MRSA HN5	2	4	4	4	8
MRSA HN M3	4	8	8	8	2
MRSA HN M4	4	4	8	4	2
MRSA HN M5	2	4	8	4	2
MRSA HN M6	1	8	16	8	8
MIC_50_	2	4	8	4	4
MIC_90_	4	8	16	8	8

### The Flavonoids From Licorice Exhibited Low Cytotoxicity to Mammalian Cells Without Hemolytic Activity

**FIGURE 3:**
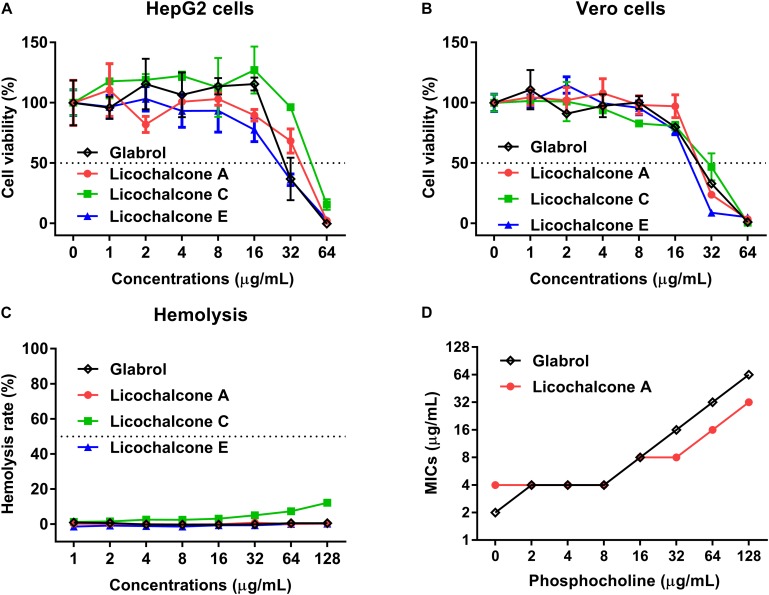
Glabrol, licochalcone A, licochalcone C, and licochalcone E exhibited low cytotoxicity for mammalian cells. **(A)** HepG2 and **(B)** Vero cells were treated with glabrol, licochalcone A, licochalcone C, and licochalcone E for 24 h, and then the cell viability was determined. **(C)** Hemolysis of glabrol, licochalcone A, licochalcone C, and licochalcone E on sheep red blood cells. **(D)** MIC of glabrol and licochalcone A with phosphocholine (PC) against *S. aureus* ATCC29213.

The cytotoxicity and hemolytics of glabrol, licochalcone A, licochalcone C, and licochalcone E were evaluated (Figure 3). The IC_50_ of glabrol, licochalcone A, licochalcone C, and licochalcone E to HepG2 and Vero cells were 31.6 and 25.2 μg/mL, 36.6 and 26.9 μg/mL, 50.8 and 27.7 μg/mL, and 25.2 and 20.4 μg/mL, respectively. Glabrol, licochalcone A, licochalcone C, and licochalcone E significantly inhibited the viability of HepG2 cells at 32–64 μg/mL and inhibited the viability of Vero cells at 16–64 μg/mL. Glabrol, licochalcone A, and licochalcone E did not cause any lysis of RBC up to 128 μg/mL, but nearly 10% of RBC were lysed by licochalcone C at 128 μg/mL. In addition, the Hly_50_ of glabrol, licochalcone A, licochalcone C, and licochalcone E were > 128 μg/mL. Moreover, phosphocholine (PC), the main phospholipid of mammalian cell membranes, inhibited the antibacterial activities of glabrol against MSSA ATCC29213 (Figure 3D).

### Glabrol Displayed Rapidly Bactericidal Activity Against MRSA and MSSA

**FIGURE 4:**
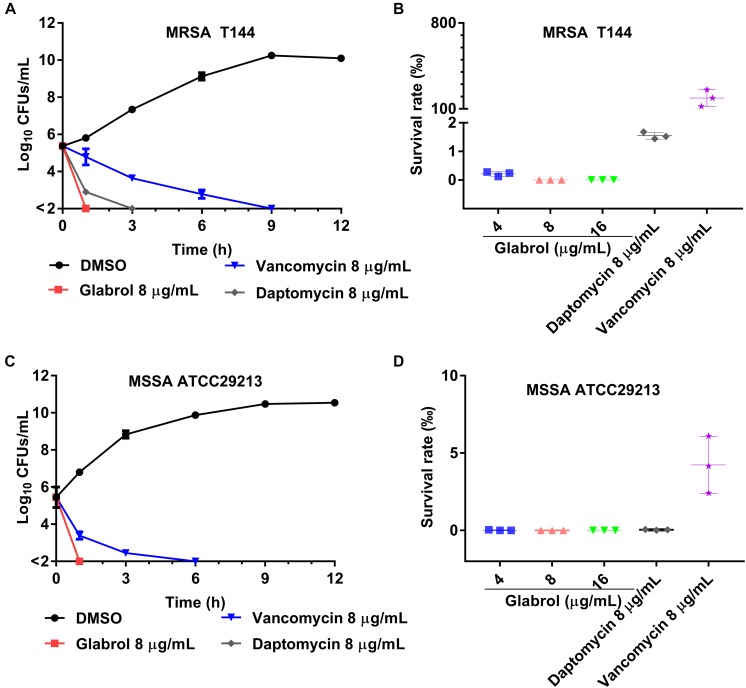
Glabrol displayed rapidly bactericidal effects against MRSA and MSSA. **(A)** MRSA T144 at exponential phase were incubated with glabrol in MHB, and then the number of viable cells was determined at the indicated time. **(B)** The cell viability of MRSA T144 at exponential phase in the presence of glabrol for 1 h. **(C)** MSSA ATCC29213 cells at exponential phase were incubated with glabrol in MHB, and then the number of viable cells was determined at the indicated time. **(D)** The cell viability of MSSA ATCC29213 at exponential phase in the presence of glabrol for 1 h. Data are presented as the means ± SD.

The cell viability of *S. aureus* cells exposed to glabrol, daptomycin and vancomycin for different times were monitored (Figure 4). In time-kill experiments, glabrol and daptomycin at 8 μg/mL killed both MRSA T144 and MSSA ATCC29213 completely within 3 h, whereas small fractions of MRSA T144 and MSSA ATCC29213 were still alive after exposure to vancomycin for 3 h. Moreover, all MRSA T144 and MSSA ATCC29213 cells were killed after exposure to glabrol at 4–16 μg/mL for 1 h, whereas nearly 5% of MRSA T144 and 10% of MSSA ATCC29213 cells survived after exposure to vancomycin for 1 h.

### Glabrol Rapidly Dissipated the Proton Motive Force and Increased the Membrane Permeability of *S. aureus*

**FIGURE 5:**
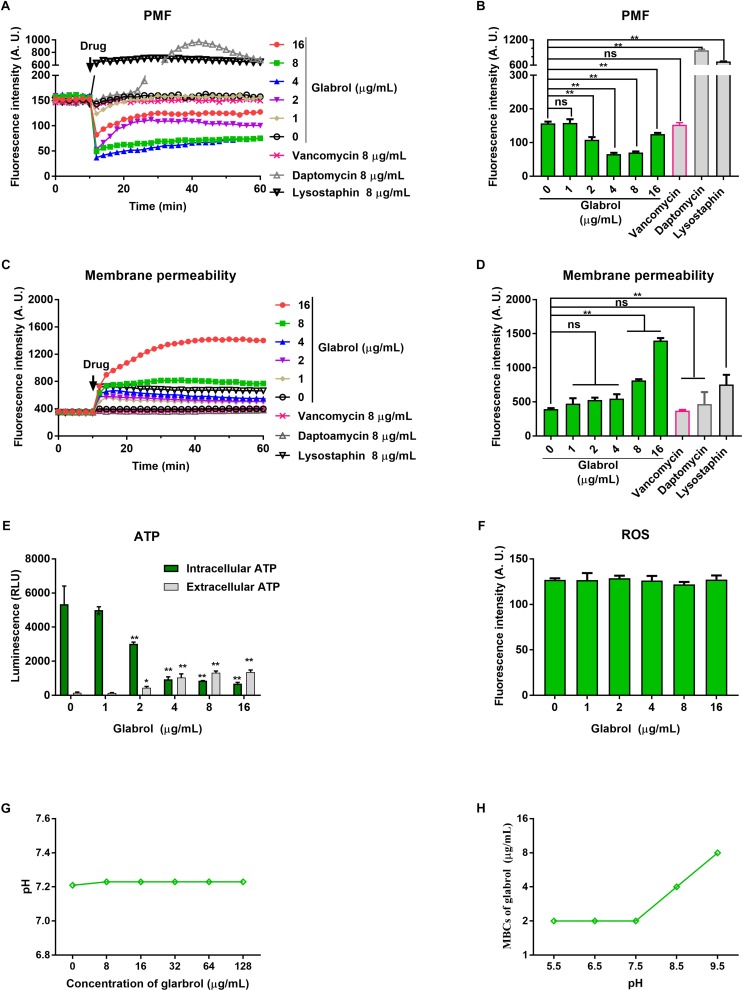
Glabrol rapidly dissipated the proton motive force and increased the membrane permeability of *S. aureus.* **(A)** *S. aureus* ATCC29213 cells were treated with DiSC3(5) for 10 min, and then treated with glabrol for 50 min. The fluorescence of DiSC3(5) was excited at 622 nm with an emission at 670 nm. **(B)** DiSC3(5) fluorescence intensity of *S. aureus* ATCC29213 cells exposed to glabrol for 30 min. Data are presented as the means ± SD. ^∗^*P* < 0.05, ^∗∗^*P* < 0.01. **(C)** *S. aureus* ATCC29213 cells were treated with PI for 10 min, and then treated with glabrol for 50 min. The fluorescence of PI was excited at 535 nm with an emission at 615 nm. **(D)** PI fluorescence intensity of *S. aureus* ATCC29213 cells exposed to glabrol for 30 min. Data are presented as the means ± SD. ^∗^*P* < 0.05, ^∗∗^*P* < 0.01. **(E)** *S. aureus* ATCC29213 cells were treated with glabrol for 1 h, then the levels of extracellular ATP and the intracellular ATP were measured. Data are presented as the means ± SD. ^∗^*P* < 0.05, ^∗∗^*P* < 0.01. **(F)** *S. aureus* ATCC29213 cells were treated with glabrol for 1 h, then the total ROS were measured with DCFH-DA. Data are presented as the means ± SD. ^∗^*P* < 0.05, ^∗∗^*P* < 0.01. **(G)** Glabrol did not alter the pH of HEPES buffer. **(H)** The antibacterial activity of glabrol decreased with the higher pH.

**FIGURE 6:**
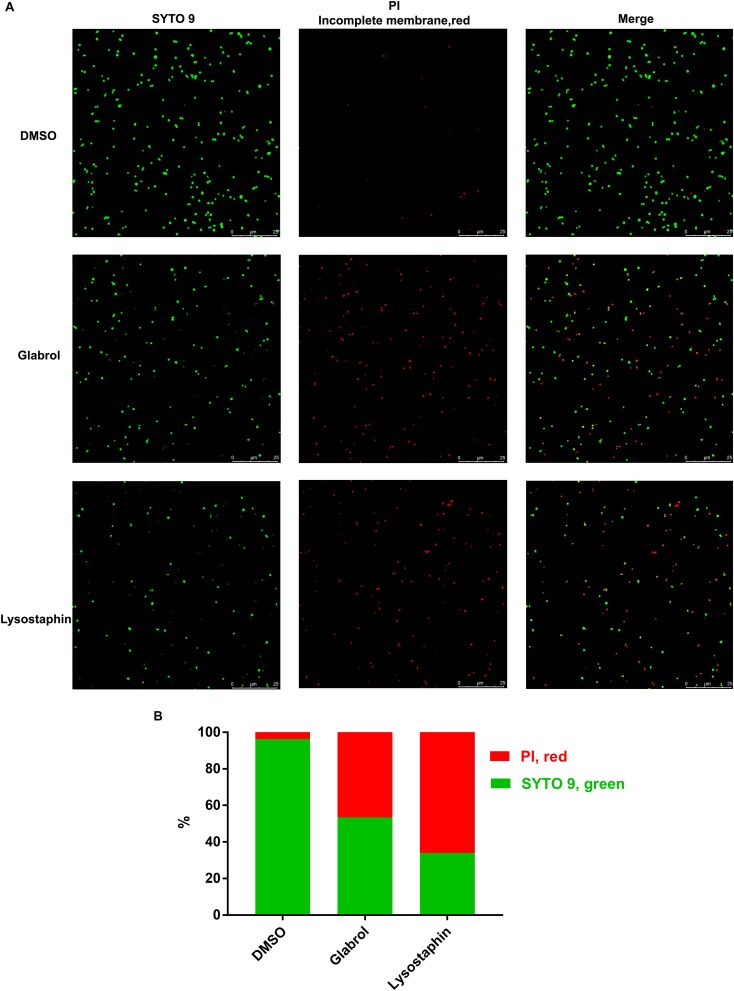
Glabrol disrupted the bacterial membrane of *S. aureus*. *S. aureus* ATCC29213 cells were treated with glabrol or lysostaphin at the concentration of 8 μg/mL for 30 min and then incubated with PI and SYTO 9. **(A)** Images captured by confocal microscopy. **(B)** The percentages of PI-labeled *S. aureus* (red) and SYTO 9-labeled *S. aureus* (green).

We next used a series of fluorescent dyes to uncover the mechanism of glabrol against *S. aureus* (Figure 5). DiSC3(5) was used to detect the change of proton movie force (PMF) containing of the membrane potential (Δψ) and the transmembrane proton gradient (ΔpH). The addition of glabrol led to a rapid change in the fluorescence intensity of DiSC3(5)-treated *S. aureus*, whereas the addition of lysostaphin and daptomycin rapidly increased the fluorescence intensity of DiSC3(5)-treated *S. aureus.* Moreover, the addition of vancomycin showed no effect on the fluorescence intensity of DiSC3(5)-treated *S. aureus*. Interestingly, after treatment for 30 min, glabrol at 2, 4, and 8 μg/mL significantly decreased the fluorescence intensity of DiSC3(5), whereas the fluorescence intensity of DiSC3(5) was not affected by glabrol at 16 μg/mL. To further assess the effect of glabrol on cytoplasmic membrane permeability, cytoplasmic membrane permeability was assessed using the dye PI. Glabrol (8–16 μg/mL) and lysostaphin rapidly increased the fluorescence intensity of PI, whereas daptomycin and vancomycin did not affect the fluorescence intensity of PI. The disruption of cytoplasmic membrane permeability and PMF would interfere with cellular ATP levels. We observed that glabrol at 2–16 μg/mL significantly increased the extracellular ATP levels and decreased the intracellular ATP levels. The levels of intracellular ROS were determined with DCFH2-DA. However, ROS production in *S. aureus* was not affected by glabrol at 1–16 μg/mL (Figure 4D). To investigate whether or not PMF is responsible for the antibacterial activity of glabrol, the MBCs of glabrol at different pH values were determined. The MBCs of glabrol at pH 5.5, 6.5, 7.5, and 9.5 were 2, 2, 2, 4, and 8 μg/mL, respectively. A confocal microscopy assay showed that glabrol and lysostaphin disrupted the membrane of *S. aureus* after incubation for 30 min (Figure 6).

### Peptidoglycan, PG and CL Inhibited the Antibacterial Effect of Glabrol Against *S. aureus*

**FIGURE 7:**
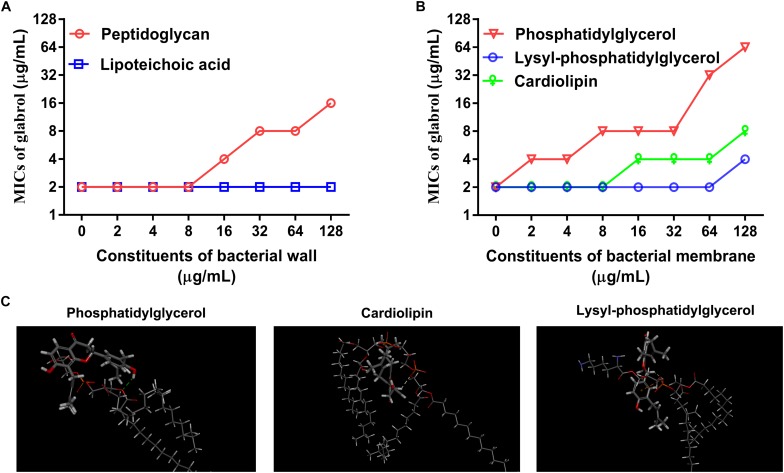
Inhibitory effect of peptidoglycan, phosphatidylglycerol, and cardiolipin on the antibacterial activity of glabrol against *S. aureus.* **(A)** Inhibitory effect of peptidoglycan on the antibacterial activity of glabrol against *S. aureus* ATCC29213. **(B)** Inhibitory effect of phosphatidylglycerol (PG), and cardiolipin (CAL) on the antibacterial activity of glabrol against *S. aureus* ATCC29213. **(C)** The interaction models of glabrol with PG, CAL, and Lysyl-PG by molecular docking with Discovery Studio Client 2018.

Having identified potential mode of action of glabrol against *S. aureus* possibly *via* the disruption of membrane function, we attempted to identify the targets of glabrol. First, we investigated the effect of peptidoglycan and lipoteichoic acids on the antibacterial activity of glabrol (Figure 7A). As shown in Figure 7, the addition of peptidoglycan increased the MIC of glabrol against MSSA ATCC29213, whereas the MIC of glabrol against MSSA ATCC29213 was not affected by lipoteichoic acids. Next, we investigated whether bacterial membrane phospholipids affect the antibacterial activity of glabrol (Figure 7B). The addition of PG and CAL resulted in an increase in the MIC of glabrol against MSSA ATCC29213. In contrast, the MIC of glabrol against MSSA ATCC29213 was not affected by lysyl-PG at 1–64 μg/mL. Finally, a molecular docking method was used to investigate the interaction models of glabrol with phospholipids. As shown in Figure 7C, glabrol binds to PG and CAL via hydrogen bonds. The interaction energies of glabrol with PG, CAL, and lysyl-PG were −20.550, −26.144, and −17.346 kcal/mol, respectively (Table 3).

**TABLE 3:** CDOCKER interaction energy of glabrol and bacterial membrane phospholipids.

**Phospholipid**	**CDOCKER interaction energy (kcal/mol)**	**Type of bonding**
PG	−20.550	Hydrogen bond
CAL	−26.144	Hydrogen bond
Lysyl-PG	−17.346	Hydrogen bond, electrostatic

**PG, phosphatidylglycerol; CAL, cardiolipin; Lysyl-PG, lysyl-phosphatidylglycerol.**

### Glabrol Displays Low Levels of Resistance Development

**FIGURE 8:**
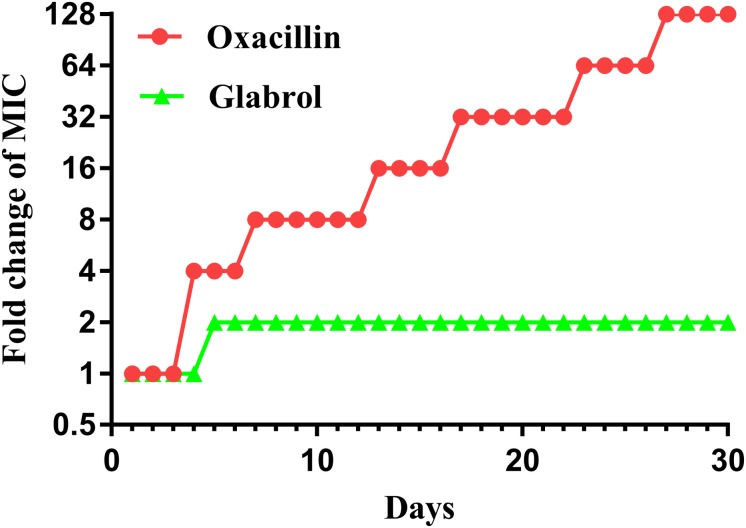
Bacterial resistance studies of glabrol against *S. aureus* ATCC29213. *S. aureus* ATCC29213 were cultured in MHB with glabrol and oxacillin at 0.5 × MIC for 24 h, and the MICs of glabrol and oxacillin were determined. The process was repeated for 30 passages.

MSSA ATCC29213 was used to study the resistance development of glabrol and oxacillin (Figure 8). There was a twofold enhancement in the MIC of glabrol after 30 passages. In contrast, the MIC for oxacillin was increased by 128-fold after 30 passages.

### Glabrol Protected *Galleria mellonella* Larvae Against MRSA

**FIGURE 9:**
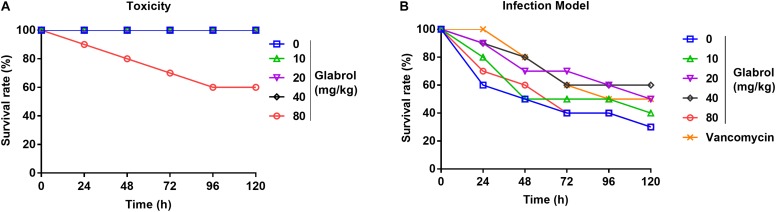
Glabrol protected *Galleria mellonella* larvae against MRSA. **(A)** *G. mellonella* larvae were treated with glabrol by injection, and the survival number of larvae was monitored for 5 days. **(B)** *G. mellonella* larvae were infected with MRSA T144 and then treated with glabrol by injection. Larvae were monitored for 5 days, and the survival number was recorded.

To evaluate the effect of glabrol on *G. mellonella* larvae infected with MRSA, the toxicity of glabrol to the larvae was first investigated. The administration of glabrol at 80 mg/kg resulted in the death of the larvae, whereas glabrol at 10, 20, 40 mg/kg did not cause the death of the larvae (Figure 9A). A model of larvae infected with MRSA T144 was used to evaluate the effect of glabrol *in vivo*. Glabrol at doses of 10, 20, and 40 mg/kg increased the survival of larvae infected with MRSA T144. At 120 h postinfection, the survival rates of glabrol at the doses of 10, 20, and 40 mg/kg at 5 days postinfection were 40, 50, and 60%, respectively, whereas the survival rate of the MRSA T144 infection group was 30% (Figure 9B).

### Glabrol Rapidly Disinfected MRSA on Cooking Utensils

**FIGURE 10:**
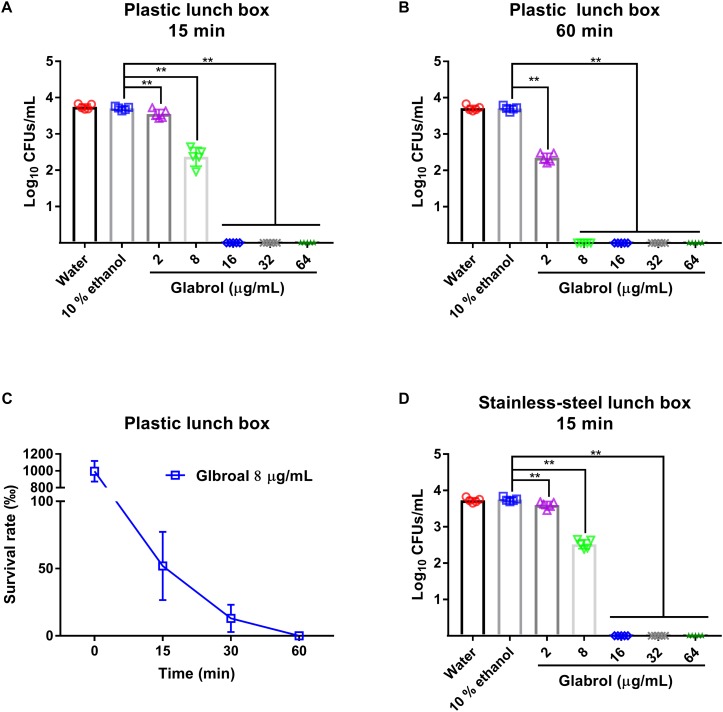
Disinfection efficacy of glabrol against MRSA on cooking utensils. **(A,B)** The plastic lunch boxes contaminated with MRSA T144 were treated with glabrol for 15 or 60 min, and then the bacteria were recovered to determine the survival number by the plate method. **(C)** The plastic lunch boxes contaminated with MRSA T144 were treated with glabrol at the concentration of 8 μg/mL, and the bacteria were recovered to determine the survival number by the plate method at the indicated time. **(D)** The stainless-steel lunch boxes contaminated with MRSA T144 were treated with glabrol for 15 min, and the bacteria were recovered to determine the survival number by the plate method. Data are presented as the means ± SD. ^∗^*P* < 0.05, ^∗∗^*P* < 0.01.

To evaluate the suitability of glabrol as a sanitizer for cooking utensils, the disinfection effect of glabrol on lunch boxes containing MRSA was investigated (Figure 10). Glabrol at concentrations of 16–64 μg/mL eliminated all MRSA T144 on the plastic lunch boxes and the stainless-steel lunch boxes within 15 min. Moreover, glabrol at a concentration of 8 μg/mL eliminated all MRSA T144 on the plastic lunch boxes and the stainless-steel lunch boxes within 1 h.

## Discussion

Medicinal plants are an important source for the discovery of potential new agents to control pathogens (Shuai-Cheng et al., 2019). In the present study, we found that glabrol, licochalcone A, licochalcone C, and licochalcone E displayed high efficiency against *S. aureus*, with low cell cytotoxicity to mammalian cells. Glabrol exerts its bactericidal effect on *S. aureus* possibly via disruption of the PMF and membrane permeability. Moreover, glabrol rapidly eliminated MRSA T144 on the plastic lunch box and the stainless-steel lunch box. Such evidence demonstrated that glabrol was one membrane-active antibacterial agent against MRSA.

Licorice has been reported to treat infectious diseases caused by MRSA (Long et al., 2013), *Pseudomonas aeruginosa* (Chakotiya et al., 2017), *Helicobacter pylori* (Han et al., 2015), *Aeromonas hydrophila* (Tang et al., 2014), and phytopathogenic fungi (Schuster et al., 2010). Licochalcone A and Licochalcone E inhibited the secretion of alpha-toxin enterotoxins A and B by *S. aureus*, which play an important role in pathogenesis (Qiu et al., 2010a, b; Zhou et al., 2012). In the present study, we found that glabrol, licochalcone A, licochalcone C, and licochalcone E displayed high efficiency against *S. aureus* (Figure 2). However, glabrol, licochalcone A, licochalcone C, and licochalcone E also exhibited low cytotoxicity to mammalian cells (Figure 3). Several reports have shown that glabrol inhibits the cancer targets cytochrome P450 1B1 enzyme (Sharma et al., 2017) and isoprenylcysteine carboxyl methyltransferase (Buchanan et al., 2008). Licochalcone A inhibits the growth of human hepatoma cells and glioma stem cells via induction of cell apoptosis, cell cycle arrest, and mitochondrial dysfunction (Kuramoto et al., 2017). Phosphocholine is the major phospholipid of mammalian cell membranes (Dias et al., 2018). The inhibitory effect of phosphocholine on the antibacterial effect of glabrol led us to speculate that the cell cytotoxicity of glabrol and licochalcone A may be due, at least partially, to a perturbation of mammalian cell membranes (Figure 3E).

The time taken for an antibacterial agent to kill bacteria provides important information on its mode of action. Membrane-active bactericides are generally bactericidal within minutes due to the interactions with bacterial membranes. The rapid bactericidal activity of glabrol possibly resulted from its membrane-targeting action (Figure 4). The PMF is composed of the membrane potential (Δψ) and transmembrane proton gradient (ΔpH), and perturbations to either Δψ or ΔpH will lead to compensatory increases in the other (Farha et al., 2013). The probe DiSC3(5) can accumulate in the cytoplasmic membrane, and the accumulation occurs in a Δψ-dependent manner. *S. aureus* exposed to daptomycin resulted in a decrease in the membrane potential Δψ, as evidenced by the higher levels of DiSC3(5) fluorescence intensity (Alborn et al., 1991; Silverman et al., 2003). Interestingly, glabrol collapsed both Δψ and ΔpH (Figures 6A,B), similar to carbonyl cyanide 3-chlorophenylhydrazone (Lamsa et al., 2012). Many antimicrobial agents collapse the PMF by increasing the cytoplasmic membrane permeability (Wilmes et al., 2011). PI binds to nucleic acids of bacteria with incomplete cytoplasmic membranes, causing an increase in fluorescence (21). Glabrol and lysostaphin rapidly induced damage to bacterial membrane in *S. aureus* cells, as evidenced by the increased fluorescence intensity of PI (Figures 5C,D). Daptomycin clusters fluid lipid domains but does not cause membrane invaginations (Muller et al., 2016), consistent with our results. Because Δψ and ΔpH are interdependent, a shift of the extracellular pH to alkaline values leads to Δψ becoming the dominant component of the PMF, and a shift of the extracellular pH to acidic values leads to ΔpH becoming the dominant component of the PMF. The poor effect of pH on the MBCs of glabrol suggested that the antibacterial activity of glabrol against *S. aureus* is more likely due to the disruption of membrane permeability (Figure 5H).

The binding of glabrol to the cell wall or the cytoplasmic membrane is important for its action on the bacterial membrane. The main components of the cell wall are peptidoglycan and lipoteichoic acids. However, the cell wall is not a good permeability barrier due to the many pores that allow many antibiotics across (Nikaido, 2003). The inhibitory effect of peptidoglycan on the antibacterial activity of glabrol supported that glabrol first binds to the peptidoglycan of *S. aureus* (Figure 7A). Phospholipids are the major components of the bacterial cytoplasmic membrane, and the most prominent phospholipids in *S. aureus* are PG, lysyl-PG, and CAL (Kuhn et al., 2015). Inhibition assays provided compelling evidence that glabrol selectively binds to PG and CAL, confirming that glabrol is a membrane-active antibacterial agent (Figure 7B). In contrast, premixing glabrol with lysyl-PG showed a poor effect on the antibacterial activity of glabrol. Molecular docking studies showed that the interaction energy of glabrol with PG and CAL were lower than that with lysyl-PG, suggesting that the binding affinities of glabrol with PG and CAL was greater than that with lysyl-PG. The mode of action of glabrol represents one new type of antibacterial agent to avoid resistance development (Figure 8).

Although glabrol exhibited a protective effect against MRSA in *G. mellonella* larvae, its application for systemic infections should be limited due to its toxicity, as evidenced by its cytotoxicity on mammalian cells and toxicity to *G. mellonella* larvae (Figures 3, 9). The selectivity of glabrol against MRSA should be improved to avoid its side effect and to improve antibacterial activity (Zou et al., 2013; Koh et al., 2015). Herbs and their extracts have been used as disinfectants due to their antimicrobial properties and low resistance development (Chen, 2004; Chandrappa et al., 2015; Anand et al., 2016). Disinfection of cooking utensils with herbal extracts is an effective strategy for the prevention of food-borne diseases. The good disinfection efficacy of glabrol against MRSA on the surface of the lunch boxes supported its use as a disinfectant candidate for the prevention of *S. aureus* (Figure 10). Furthermore, its poor selectivity and low solubility in water remain to be addressed.

In summary, our data demonstrated that flavonoids from licorice, including glabrol, licochalcone A, licochalcone C, and licochalcone E, displayed good antibacterial activities against MRSA with low cytotoxicity to mammalian cells. Glabrol rapidly disrupted the PMF and membrane permeability of *S. aureus* possibly by binding to peptidoglycan, PG and CAL. Glabrol protected *G. mellonella* larvae against MRSA with low systemic toxicity. Moreover, glabrol exhibited a good disinfection effect on MRSA on the plastic lunch boxes and on the stainless-steel lunch boxes. Taken together, glabrol is a potential lead compound for the design of membrane-active drugs against MRSA that are based on flavonoids and can be used as a disinfectant candidate for cooking utensils.

## Data Availability Statement

The raw data supporting the conclusions of this manuscript will be made available by the authors, without undue reservation, to any qualified researcher.

## Ethics Statement

The protocol was approved by the Ethical Committee for Institutional Animal Use and Care of China Agricultural University.

## Author Contributions

KZ and J-ZS conceived and designed the study. S-CW, QL, Z-QY, and FL designed and performed the experiments. S-CW, W-JP, S-QQ, and X-BS collected and analyzed the experimental data. S-CW and KZ wrote the manuscript. All authors reviewed the manuscript.

## Conflict of Interest

The authors declare that the research was conducted in the absence of any commercial or financial relationships that could be construed as a potential conflict of interest.
